# TurkPrime.com: A versatile crowdsourcing data acquisition platform for the behavioral sciences

**DOI:** 10.3758/s13428-016-0727-z

**Published:** 2016-04-12

**Authors:** Leib Litman, Jonathan Robinson, Tzvi Abberbock

**Affiliations:** 1 Lander College, Flushing, New York USA; 2 Department of Psychology, Lander College, 75-31 150th Street, Flushing, New York 11367 USA

**Keywords:** Mechanical Turk, Crowdsourcing, Online research

## Abstract

In recent years, Mechanical Turk (MTurk) has revolutionized social science by providing a way to collect behavioral data with unprecedented speed and efficiency. However, MTurk was not intended to be a research tool, and many common research tasks are difficult and time-consuming to implement as a result. TurkPrime was designed as a research platform that integrates with MTurk and supports tasks that are common to the social and behavioral sciences. Like MTurk, TurkPrime is an Internet-based platform that runs on any browser and does not require any downloads or installation. Tasks that can be implemented with TurkPrime include: excluding participants on the basis of previous participation, longitudinal studies, making changes to a study while it is running, automating the approval process, increasing the speed of data collection, sending bulk e-mails and bonuses, enhancing communication with participants, monitoring dropout and engagement rates, providing enhanced sampling options, and many others. This article describes how TurkPrime saves time and resources, improves data quality, and allows researchers to design and implement studies that were previously very difficult or impossible to carry out on MTurk. TurkPrime is designed as a research tool whose aim is to improve the quality of the crowdsourcing data collection process. Various features have been and continue to be implemented on the basis of feedback from the research community. TurkPrime is a free research platform.

Access to participants is of central importance to researchers in the social and behavioral sciences. Until the late 1990s, most behavioral researchers had to rely on undergraduate subject pools as their primary source of research participants. University subject pools have a number of limitations, including lack of representativeness (Henrich, Heine, & Norenzayan, 2010), and can often be labor-intensive and time-consuming to use. Most importantly, such subject pools are not available to faculty in many small schools (see Kraut et al., 2004). Starting in the late 1990s, the Internet became more commonly used as a source of participant recruitment (Kraut et al., 2004). Recruiting participants over the Internet provided a number of advantages over the use of the traditional subject pool (Gosling & Johnson, 2010): Researchers were able to recruit vastly more participants in much shorter time periods (Nosek, Banaji, & Greenwald, 2002), and the data were typically more representative and less labor-intensive to acquire (Kraut et al., 2004). Accessing large numbers of participants from the Internet, commonly referred to as *crowdsourcing*, also has substantial limitations. For example, finding participants, incentivizing participation, and preventing multiple participation are just some of the common barriers encountered on most crowdsourcing platforms.

One crowdsourcing platform that overcomes many of these limitations is Amazon Mechanical Turk (MTurk). MTurk has in recent years revolutionized behavioral research (Buhrmester, Kwang, & Gosling, 2011), and increasingly is being used as a participant recruitment tool across a wide spectrum of disciplines in the social sciences. MTurk is an online platform where researchers can post studies, instantaneously making them available to thousands of participants around the world. Hundreds of participants will typically have completed a study just hours after it is launched, drastically increasing the speed of the data acquisition process relative to traditional methods. Data acquired on MTurk have been found to be valid across numerous tasks and countries (Litman, Robinson, & Rosenzweig, 2015; Shapiro, Chandler, & Mueller, 2013; Sprouse, 2011), despite the relatively low price that participants are paid for completing tasks, making the MTurk platform a fast method of acquiring cheap and reliable data (Buhrmester et al., 2011).

Despite the benefits that MTurk offers social science researchers, it also has its limitations. MTurk was not created with behavioral research in mind, and many features that are fundamental for the research process are not readily available through the MTurk graphical user interface (GUI). To overcome the current limitations of MTurk, multiple tools have been created to enable researches to accomplish specific tasks (Gureckis et al., 2015; Peer, Paolacci, Chandler, & Mueller, 2012; http://gideongoldin.github.io/TurkGate/). For example, Peer et al. (2012) created a Qualtrics tool through which it is possible to exclude participants from a study on the basis of participation in previous studies. However, currently no single, integrated platform provides researchers with a comprehensive set of research-specific flexible tools that are delivered through a simple GUI. For this reason, we developed the TurkPrime Data Acquisition Platform for the Social Sciences (TurkPrime). TurkPrime is a website on which users can create MTurk studies (HITs) and through which MTurk HITs can be controlled with substantially greater flexibility than the MTurk platform currently allows.

TurkPrime (see Fig. 1) is a website that utilizes the MTurk application programming interface (API), as well as other programmatic tools, to provide MTurk requesters with a GUI environment with a high level of control over HITs. The TurkPrime GUI environment provides improved functionality over MTurk in six general areas: control over who participates in the study, flexible control over running HITs, more flexible communication and payment mechanisms, tools for longitudinal and panel studies, tools to increase sample representativeness, and enhanced study flow indicators.

**Fig. 1:**
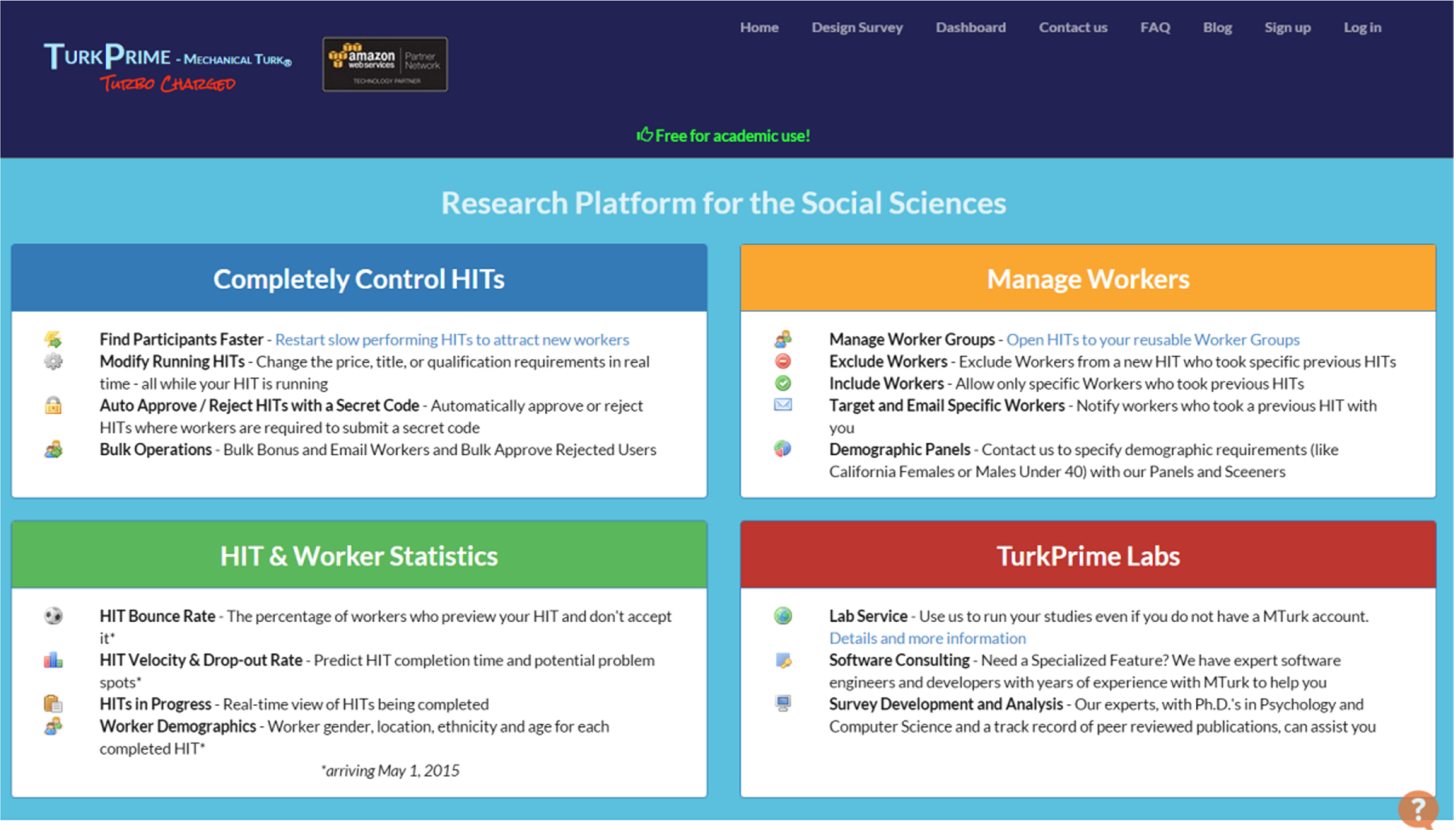
Screenshot of the TurkPrime home page

In the present article, we show how the TurkPrime environment allows users to overcome the current limitations of the MTurk GUI. Because detailed tutorials including videos for using TurkPrime are available on the TurkPrime website, in this article we focus on discussing the methodological advantages that TurkPrime offers, and also provide a conceptual overview of the TurkPrime platform.

## Mechanical Turk basics

Because of MTurk’s popularity, a number of MTurk guides are currently available (Berinsky, Huber, & Lenz, 2012; Chandler & Mueller, 2013; Paolacci, Chandler, & Ipeirotis, 2010; Mason & Suri, 2012). Here, we will not provide a comprehensive overview of MTurk, but only introduce those terms and concepts that are relevant for the TurkPrime features that are discussed throughout this article.

### Workers, requesters, and HITs

The MTurk platform was created as a worldwide microtask marketplace. MTurk facilitates monetary transactions between people who need various jobs done (*requesters*) and people who are interested in performing those jobs in return for payment (*workers*). A task put up by a requester on MTurk is called a *human intelligence task* (*HIT*). Requesters can create HITs on the MTurk platform or use MTurk to announce an external HIT. For example, MTurk provides a platform for creating survey questions. A requester may use that built-in MTurk functionality, or instead can create a survey on an external platform such as Qualtrics and post a link to that survey as part of their HIT. We refer to HITs that are completed via an external platform as *external HITs*, and tasks that utilize the MTurk platform as *internal HITs*.

MTurk makes HITs visible to workers by listing them in the All HITs window, where workers can select a HIT from the numerous ones available. Workers can organize HITs in various ways. By default, the All HITs window organizes HITs by their creation date, such that the newest HITs are presented at the top of the list. HITs can also be sorted by price, title, and HIT length.

### Worker ID

The MTurk platform allows for two-way interactions between workers and requesters. For example, requesters can send e-mail invitations to workers and issue bonuses based on performance. Requesters can also issue qualifications to workers. A qualification is a status indicator that reflects whether or not a worker is eligible to take a specific HIT with a requester. By using Worker IDs, MTurk prevents workers from participating multiple times in the same study. This is one of the critical features that makes data collection on MTurk different from regular online data platforms, where there is typically little control over a participant’s ability to complete a study multiple times.

When signing up for an account, each worker is assigned a unique Worker ID. This Worker ID cannot be changed and remains constant as long as the worker uses that account. The Worker ID is key for the overall MTurk functionality for multiple reasons. For example, by using a worker’s ID, a requester may invite a specific worker to take a follow-up HIT or send a monetary bonus to that worker.

### Approving and rejecting HITs

MTurk keeps track of workers’ HIT histories and work quality by means of a number of indicators. Requesters can use these indicators as selection criteria when specifying the eligibility of workers for taking a HIT. When workers complete a study, their Worker IDs automatically appear in a list of completed requester’s HITs. A requester can *approve* the HIT, at which point the payment for that HIT is made automatically by MTurk from the requester’s funded MTurk account to the account of the worker. A requester can also *reject* a HIT if it was not performed adequately, for whatever reason. Overall, the requester has a lot of control over the worker and has deciding power over whether or not a HIT is rejected. A rejected HIT results in a worker not being paid. Additionally, the numbers of approved and rejected HITs remain part of a worker’s permanent record. The ratio of approvals to rejections makes up a worker’s a*pproval rating*, which can be used by a requester at the beginning of a study to select workers. Selecting workers with high approval ratings has been shown to improve data quality (Peer, Vosgerau, & Acquisti, 2014).

A worker’s approval rating is updated every time the worker finishes a study and is either rejected or approved by a requester. The reciprocal relationship, whereby workers would rate requesters, is not provided by MTurk. However, various websites on which workers leave comments about specific HITs and requesters, such as Turkopticon (https://turkopticon.ucsd.edu/) and TurkerNation (http://turkernation.com/), have been created, in part to provide workers with information about the quality of requesters and HITs. Workers commonly provide information on these websites about whether a HIT pays adequately, how long a requester takes to pay for a HIT, and whether a requester rejects HITs unfairly. Requesters should keep track of these websites, because information provided there can affect workers’ willingness to accept a specific requester’s HIT. Additionally, requesters should manage their reputations on these sites, because the information provided there is not always accurate, and can potentially negatively affect participation rate, and thus may compromise the recruitment process.

### Monitoring worker quality

In addition to a worker’s approval rating, MTurk also keeps track of the number of HITs the worker has completed and the number of HITs returned. When a requester creates a HIT, it becomes visible to the MTurk worker community on the All HITs page, which lists the titles of all available HITs. A worker may click on the name of a HIT that they are interested in—for example, “transcribe data”—which opens that HIT in preview mode, in which a more detailed description of the HIT becomes visible. For an internal HIT, workers will see a preview of the task, and for an external HIT, they will see a link to the external site, and typically a more detailed description of the task.

All of the MTurk functionality described thus far can be navigated through the MTurk GUI and does not require any programming. In addition to the GUI, MTurk provides an API with which much more flexibility can be achieved over HITs. However, use of the API requires a knowledge of programming and a substantial time commitment. The result is that most requesters do not benefit from the full set of resources that MTurk offers. The standard GUI interface imposes critical restrictions on what requesters can do with their HITs. For example, they cannot easily prevent workers from participating in a study on the basis of their participation in previous studies, set up longitudinal studies, automatically approve worker assignments upon completion of a study, communicate with multiple workers at once, grant bonuses to more than one worker at a time, change the price or description of a study after it has been launched, or control the rate at which data are collected. TurkPrime was created as a way to overcome these and other limitations, for the purpose of giving requesters more control over their studies.

## Using TurkPrime

### Initial TurkPrime setup

To use TurkPrime, a requester must (a) have an existing MTurk account, (b) create a TurkPrime account, and (c) link the TurkPrime account to their MTurk account. The TurkPrime website (TurkPrime.com) has a Setup Mechanical Turk tab, which provides the directions for associating a TurkPrime account with an MTurk account. This short process involves providing TurkPrime with a requester’s MTurk account access keys, which are found on their MTurk security credentials page. Once the account is set up, requesters can launch, stop, and make changes to their MTurk HITs directly from TurkPrime.

### The TurkPrime environment

**Fig. 2:**
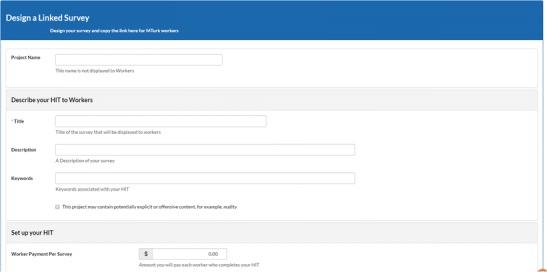
Screenshot of the Design Survey page

**Fig. 3:**
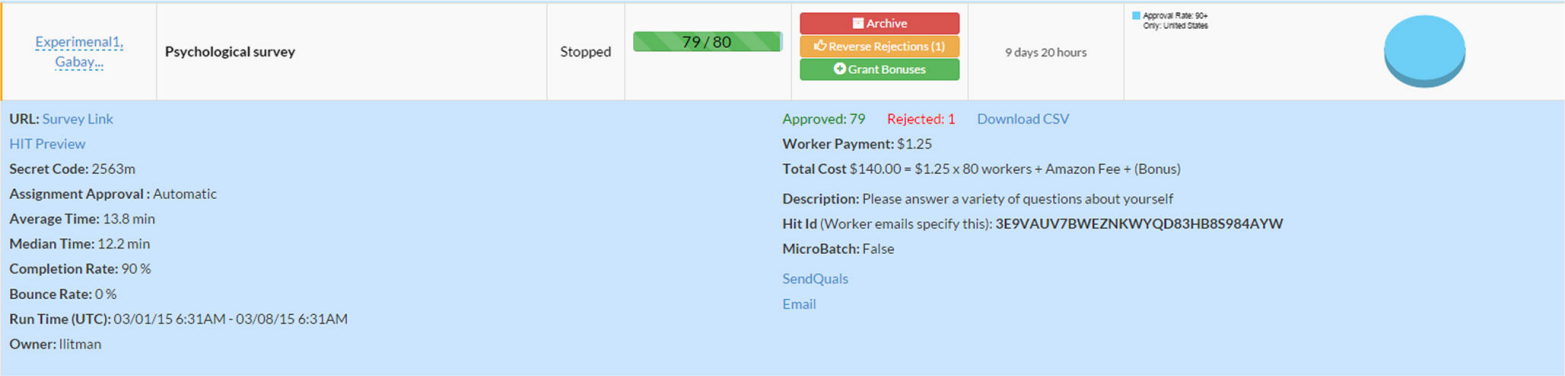
Screenshot of the Dashboard extended view

**Fig. 4:**
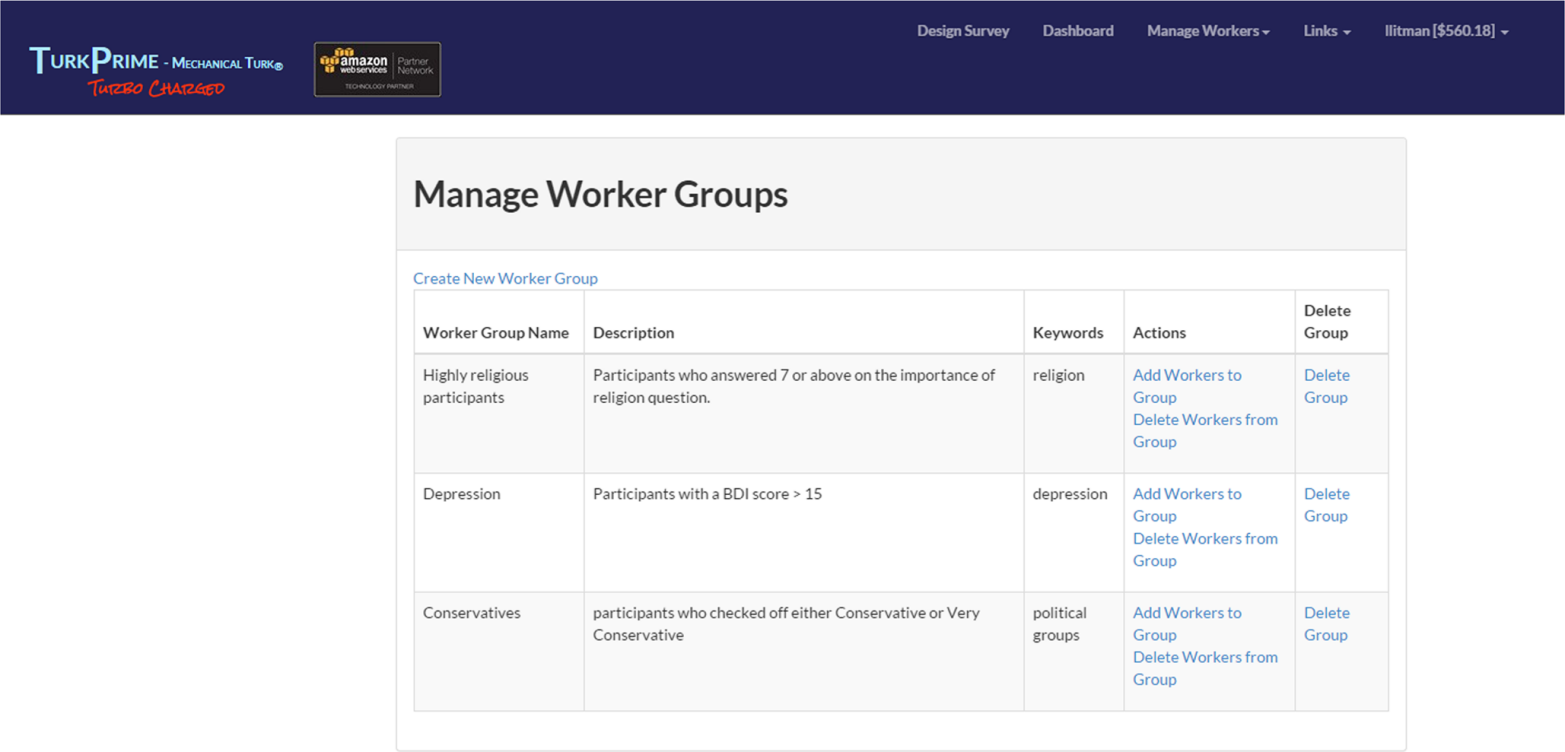
Screenshot of the Worker Groups window

TurkPrime has three primary windows. The Design Survey page (see Fig. 2) is where studies are set up and prepared for launch. The Dashboard (see Fig. 3) provides a view of all running and completed studies. It additionally contains an interface through which a study can be controlled, and indicators that provide information about the study’s progress. An additional Workers Groups (see Fig. 4) page is discussed below, in the Longitudinal and Panel Studies section. As on MTurk, TurkPrime provides a Sandbox environment in which HITs can be simulated without any actual data being collected.

### Design Survey page

TurkPrime studies are launched from the Design Survey page, which can be accessed from TurkPrime’s home page. The Design Survey page is designed to look like the Design HITs page on MTurk. Experienced MTurk users will find that setting up a study on TurkPrime is very similar to doing so on MTurk. For example, requesters will find fields for naming a study, indicating the price, and selecting the quality of workers. The MTurk Design Survey page also contains fields that are not found on MTurk, such as excluding or including participants from prior studies (see Fig. 4).

### Control over who participates in a study

TurkPrime provides a number of ways to limit and grant access to a study for specific workers. In effect, TurkPrime automates the process of assigning qualifications. TurkPrime makes it possible to assign qualifications to participants from multiple studies simply by selecting those studies from a dropdown window or pasting sets of Worker IDs in the Include and Exclude windows.

#### Excluding workers

Allowing participants to complete a study multiple times can lead to a reduction in effect sizes (Chandler, Paolacci, Peer, Mueller, & Ratliff, 2015). It is thus important to be able to exclude participants from a HIT on the basis of prior participation in a similar HIT. On TurkPrime, participants can be excluded from a HIT in two ways. The first is to enter their Worker IDs into an Exclude box when launching a HIT. One can enter any Worker ID in this box, even if the Worker IDs are from a HIT that was not run on TurkPrime, provided that these workers have an active relationship with a requester. The second way to exclude participants is at a study level. When creating a new HIT (e.g., HIT 4), a requester may select a whole HIT (HIT 1) or HITs (HIT 1, HIT 2, HIT 3) for exclusion from HIT 4, using the dropdown menu in the Exclude Workers Who Completed These Surveys window. This option requires that the HITs were run on TurkPrime. It is also possible to use these windows in combination to exclude both whole studies and individual workers from other studies.

A third way to exclude participants is to create worker groups. Worker Groups is a highly flexible tool with which groups can be created for exclusion and reused across multiple studies (see Fig. 4). For example, the IDs of all workers who participated in a series of studies can be added to a group. These Worker IDs can come from studies that were run on TurkPrime or that were run directly from the requester’s MTurk account. Once the group is created, it becomes visible on the Design Survey page and can be selected for exclusion. Worker groups is also useful for creating customized exclusion groups that are based on workers’ responses. For example, workers who do not pass attention manipulation checks can be added to a permanent exclude group that a requester may add to all studies. Other uses of worker groups discussed below include creating groups of experts for stimulus preparation, the creation of panels, and longitudinal studies.

#### Including workers

The Include option assigns qualifications to workers by means of which those workers, and only those workers, become eligible to take a specific HIT. The Design Survey page includes two controls through which workers can be included in a study. When setting up a study on the Design Survey page, under the Advanced tab, all of a requester’s previously launched HITs appear in the Allow Workers Who Completed These Surveys field. A requester can select multiple studies whose workers they wish to include. When including multiple studies, the “All” option includes workers who have taken all of the selected studies, and the “Any” option includes workers who have taken any of the selected studies. The Include Workers tool allows requesters to run longitudinal studies that are described in more detail below.

### Enhanced control over HITs

#### Editing a HIT after launch

TurkPrime gives requesters full control over a study after it has been launched. After launching a study, a requester can take multiple actions to control HITs that are still running and HITs that have already been completed (see Fig. 3). The Edit button takes the requester back to the Design page, where changes can be made to any of the fields, including the payment field, study description, qualifications, and any of the other fields. A requester can also pause a study and continue at a later time.

Being able to alter a study while it is running has multiple research-related uses. For example, it is often difficult to gauge the length of a study and, thus, how much to pay participants. With the Edit feature, a pilot study can be run to assess a study’s completion time. On the basis of this information, the study description and price can be altered prior to launching the full study. In effect, this allows researchers to more easily conduct pilot studies, on the basis of which a HIT’s parameters can be tuned prior to launching the full study.

#### Restart

Additional control over a running HIT is provided by the Restart button, which is found on the Dashboard. The purpose of the Restart feature is to enable requesters to collect large datasets by periodically making the HIT more visible to workers. Because thousands of HITs are available on MTurk at a given point in time, it is important to take MTurk’s approach to organizing HITs into consideration, so as to maximize the likelihood of workers seeing a HIT. For example, trivial features of a task such as its name can affect participation rates. HITs that start with the letter A or the letter Z are more likely to be taken than HITs that start with the letter S, because those are the tasks that come up first when sorting by HITs’ titles (Chilton, Horton, Miller, & Azenkot, 2010). Studies have also shown that older HITs are less likely to be viewed by workers, and that participation rates drop off very quickly for older tasks. Specifically, participation in tasks that are more than 24 h old drops off by 70% and continues to do so every consecutive day (Chilton et al., 2010). The Restart feature effectively creates a brand new HIT with characteristics identical to those of the original, and automatically prevents multiple participation by assigning qualification to workers who have taken the HIT already. The result is that the HIT is bumped up to the top of the All HITs window, which has an immediate impact on the visibility of the HIT and substantially increases participation rates.

### Flexible payment mechanisms

The receipt of timely payment is a key concern for MTurk workers. Delays in payment can result in negative reviews on Turker Nation and Turkopticon and can have negative effects on participation rates as a result. Conversely, fast payment is commonly mentioned by workers in positive reviews of HITs. The current MTurk payment mechanisms, however, can result in lengthy delays and can be time-consuming for the requester. These delays arise for a number of reasons. Requesters have to approve each worker’s submitted HIT in order for the worker to receive payment. Once a worker completes a HIT, they must wait for the requester to review and approve it. For the vast majority of HITs, the review process entails verifying what is referred to as the “secret code” that is appended to the end of a study. To verify that workers have actually completed the full HIT, requesters routinely append a short alphanumeric code at the end of their study or survey. The workers are then asked to enter this code in an MTurk response field prior to submitting their HIT. Prior to approving the HIT, requesters review the code to make sure that it is correct.

Reviewing secret codes can be time-consuming, especially for studies with large sample sizes, and can result in payment delays. To overcome this problem, TurkPrime includes two optional automatic verification mechanisms. Both mechanisms check the secret code entered by the worker against a prespecified secret code and automatically approve a submitted HIT on the basis of whether the codes match. This results in automatic and immediate payment delivery to the workers and saves the requesters time spent having to do this manually. The two verification mechanisms are called *fixed* and *dynamic* secret codes.

#### Fixed secret code

A fixed secret code is a single code entered by the requester on the Design Survey page that is the same for all workers. This code is manually added by the requester to a study, usually at the end of a survey. Workers are asked to enter this code on Mechanical Turk after they have completed the study. As we mentioned above, MTurk provides the capacity to create internal HITs. The secret code entered by workers on MTurk becomes part of this internal HIT. TurkPrime then compares the code entered by the worker against the secret code that was entered by the requester on the Design Survey page. A submitted assignment is automatically approved when there is a match. In the event of a mismatch, the assignment enters a Pending status. Pending assignments require manual review by the requester. Because static secret codes are the same for all workers, there is a possibility that such codes may be shared by workers among themselves. For this reason, it is recommended that dynamic secret codes be used when possible.

#### Dynamic secret code

A dynamic secret code works through integration with Qualtrics. The Qualtrics Survey Flow functionality can be used to display a unique code that is shown to the worker and is also automatically passed to TurkPrime. As with the fixed secret code, the dynamic secret code is entered by workers on MTurk when they complete a study. When a worker enters the dynamic secret code, it is automatically checked by TurkPrime, and workers are automatically approved and paid. Workers are not automatically rejected. In the event that a requester rejects a worker in error, the Dashboard contains a Reverse Rejections button through which rejections of individual workers or all workers can be immediately reversed.

#### Batch bonus

An additional feature for enhancing payments is the batch bonus. Bonuses are commonly used by researchers as a way to incentivize participation, which has been found to have a substantial effect on data quality and the creativity of workers (Buccafusco, Burns, Fromer, & Sprigman, 2014). The MTurk API, however, only allows for one bonus to be given to one worker at a time. This results in payment delays and can be time-consuming to implement. The TurkPrime Batch Bonus window is available on the Dashboard, through which bonuses can be assigned to all or some workers at once.

### Tools for longitudinal and panel studies

Longitudinal studies are HITs that are open to workers who have taken previous HITs with a requester. Setting up longitudinal studies requires (1) A HIT that is open only to specific workers on the basis of participation in previous studies, (2) matching participants across the phases of a study, and (3) notifying participants that a HIT is available. As we described previously, a HIT that is open only to specific workers can be set up by using the Include control.

#### Embedding study-specific fields

Because longitudinal studies have multiple phases, there are typically multiple data files that store the results from the different phases. Worker IDs allow data to be matched across phases. TurkPrime facilitates the process of matching Worker IDs across multiple data files by automatically passing each worker’s ID to Qualtrics via an embedded query string in the survey’s URL. Requesters can then automatically insert the Worker ID into data files. In Qualtrics this is easily accomplished by embedding the Worker ID field into the survey flow. As of now, this feature is only available for Qualtrics studies.

#### Worker notification

Opening up a HIT to specific workers differs from a typical HIT in a critical way. Workers who are qualified to take the HIT may not be in front of a computer at the time that a study is launched, and thus may never know that the HIT is available. For this reason, TurkPrime enables a batch e-mail feature for longitudinal studies with which e-mails can be sent to all eligible participants, informing them that the HIT is available, how much it pays, and any other information that a requester may wish to include to incentivize the workers to participate in the study. The e-mail automatically includes the HIT link.

#### Worker groups

TurkPrime has a separate Worker Groups window that enables the creation of panels, which can be considered to be a special case of longitudinal studies. Panel groups are collections of workers who share common characteristics. For example, these can be political groups such as Republicans or Democrats. Groups of Worker IDs can be entered into the Groups window and given a name. Once a group is created, it becomes visible in the Groups dropdown menu on the Design Survey page. A study can then be created that is open only to the participants of these groups. Worker group studies can be especially effective when requesters have accumulated a large number of workers that can then be utilized as a requester’s own subject pool (see Von Emden, 2015). For example, a requester can create time-series studies with equal numbers of Republican and Democratic participants, and follow them over time to examine their opinions and event-related opinion changes.

To summarize, the Include controls, worker groups, batch emails, and embedded Worker IDs constitute a set of tools that enable researchers to conduct longitudinal and time-series studies on Mechanical Turk.

### Tools for increasing sample representativeness

The speed with which data can be acquired on MTurk is one of the major benefits of this platform. However, very fast acquisition of data also has the potential for unintended negative consequences, since collecting data quickly has the potential to create bias. For example, if a study is launched at a specific time on a weekday, such as Monday morning, the sample will be biased to workers who are at home on a day when most people are working (Rosenzweig, Litman, & Robinson, 2015). To offset this potential source of bias, TurkPrime has a microbatch feature that allows requesters to break up HITs into small segments and to specify the time interval at which the segments will be launched. For example, a study may be timed to launch over 12-h periods across all seven days of the week.

Additionally, a time release feature allows requesters to specify the time that the study will be launched. With this feature, a study can be prepared at one time and launched at a later time. This is particularly useful for international requesters who launch their studies at a time that corresponds to nighttime in the USA.

### Enhanced study flow indicators

The extended Dashboard view provides a number of diagnostic tools with which a study can be monitored. These include bounce rate, completion rate, and median completion time.

#### Completion rate

Dropout rate can be an important indicator of data quality. A high dropout rate may mean the presence of a selection bias that may influence the representativeness of the results. If a HIT takes much longer to complete than is indicated in the study description, workers will be more likely to return or abandon it, influencing the dropout rate. A requester may want to modify the pay rate if the completion rate is low. A very high dropout rate may also mean that something is wrong with the study, such as a broken link that prevents participants from completing the HIT. It is typically good practice to report the completion rate (Eysenbach, 2004); however, this information is not available on MTurk. Because completion rate can be an important indicator of a study’s quality, TurkPrime makes completion rate available on the Dashboard, so that researchers can monitor the completion rate in real time.

#### Bounce rate

Bounce rate is similar to completion rate, and is important for the same reasons. What makes bounce rate different is that it also counts participants who did not accept the HIT, but merely previewed it. We define the bounce rate as 100% – [Accepted Assignments] / [Previewed Assignments]. Previewed Assignments is set to be the total of unique IP addresses that previewed this assignment. Since nearly all workers have unique IP addresses, this is a reasonable estimate of the total number of previews.

#### Completion time

The third indicator available on the extended Dashboard view is the median completion time. We have consistently observed that many workers are outliers in terms of completion times. This may be due to the fundamental lack of experimenter control over the study setting, which makes it easy for participants to take breaks in the middle of a study. These outliers affect the mean completion time. Because completion time is critical for setting appropriate compensation rates, we made the median time available on the Dashboard. We have consistently observed that median completion times are significantly lower than the means.

## Related platforms

Mechanical Turk is becoming increasingly important for data acquisition across the spectrum of behavioral research fields. At the same time, although the demand for increased versatility in the range of online research designs increases, the MTurk GUI has remained mostly unchanged. For this reason, a variety of research platforms have emerged to facilitate the use of MTurk for behavioral research. TurkPrime is one such platform, but other platforms are also available (Gureckis et al., 2015; Peer et al., 2012; http://gideongoldin.github.io/TurkGate). These platforms vary widely in the range of tools that they offer and the GUIs that they provide. One platform that offers a wide range of tools is psiTurk. Like TurkPrime, psiTurk offers the abilities to exclude participants, offer bonuses, and make automatic payments, among many other features. psiTurk is open-source platform for designing flexible online studies (see Gureckis et al., 2015). One key difference between TurkPrime and psiTurk is that psiTurk runs on a UNIX-based command line interface. As such, psiTurk only runs on UNIX-based systems, such as Linux and Mac OS. TurkPrime, on the other hand, has a point-and-click interface. TurkPrime runs on any browser from any operating system. In addition, TurkPrime provides multiple features that, to the best of our knowledge, are not currently offered on other platforms. These features include automatic checks of secret codes, including unique secret codes generated by Qualtrics; Microbatch functionality for improved sample representativeness; worker groups; the ability to easily restart a HIT; longitudinal study control, including embedding query string parameters to automatically insert a Worker ID into a data file; and full ability to modify any aspect of a HIT after it is launched.

## Usage information

### Adoption by the community

As of the writing of this article, TurkPrime has 1726 registered users who have run 9750 unique MTurk HITs. In all, over 60,000 unique workers have completed HITs on TurkPrime, and 207,000 assignments have been completed on TurkPrime within the last 30 days.

### Data protection

TurkPrime uses transport layer security encryption (also known as HTTPS) for all transmitted data. All data access is blocked except for explicitly white-listed IP addresses, in addition to being secured with user passwords. Furthermore, MTurk data, including Access Key ID and Secret Access Key, are encrypted with AES-256 encryption, the standard adopted by the National Institute of Standards and Technology.

## Conclusion

To summarize, TurkPrime was created as an extension of Amazon Mechanical Turk to optimize MTurk functionality for the needs of researchers. MTurk is a powerful crowdsourcing environment that provides substantial advantages over other online subject recruitment platforms. Among these are fast and easy access to thousands of research participants, a payment mechanism to incentivize participation, a way to prevent multiple participation by the same individual, and a high level of confidentiality. In recent years, MTurk has become the most popular crowdsourcing research platform, with close to 1,000 peer-reviewed papers published from its data per year.

The vast power of the MTurk API, however, has been considerably underutilized by most researchers. TurkPrime offers a programming-free user interface that enables researchers to harness the full power of MTurk. TurkPrime enables researchers to employ research designs, including longitudinal, time-series, and panel studies, that would otherwise be difficult and time-consuming, and that would require substantial API programming to implement. TurkPrime enhances the ease with which requesters can communicate with workers, including sending batch e-mail notifications and bonus payments; provides a flexible way to break up studies over time; gives users complete control over launched HITs; automates the approval/rejection process; provides Qualtrics integration; and allows for the creation of custom worker groups for inclusion and exclusion across studies. Worker groups have the additional benefit that requesters can notify workers who are not monitoring MTurk at the time of study launch. This makes it possible for requesters who have accumulated large worker pools to reach more workers than they would be able to by launching a study on MTurk alone.

TurkPrime was created as a dynamic environment that is responsive to the needs of researchers. As such, new features have been recommended by the research community and are being added in an ongoing effort to enhance the quality and usability of online research.
